# The COLO‐COHORT (Colorectal Cancer Cohort) study: Protocol for a multi‐centre, observational research study and development of a consent‐for‐contact research platform

**DOI:** 10.1111/codi.16160

**Published:** 2022-06-03

**Authors:** James S. Hampton, Sara Koo, Christina Dobson, Christopher J. Stewart, Laura J. Neilson, Kyle Montague, Suparna Mitra, John Whelpton, Caroline Addison, Phil Kelly, Stephen Rushton, Mark A. Hull, Linda Sharp, Colin J. Rees

**Affiliations:** ^1^ Department of Gastroenterology South Tyneside and Sunderland NHS Foundation Trust South Shields UK; ^2^ Population Health Sciences Institute Newcastle University Newcastle upon Tyne UK; ^3^ Translational and Clinical Research Institute Newcastle University Newcastle upon Tyne UK; ^4^ Computing and Information Sciences Northumbria University Newcastle upon Tyne UK; ^5^ Leeds Institute of Medical Research University of Leeds, St James's University Hospital Leeds UK; ^6^ Patient and Public Involvement Lead UK; ^7^ North East Hub Bowel Cancer Screening Programme Gateshead UK; ^8^ School of Biology Newcastle University Newcastle upon Tyne UK

**Keywords:** colorectal cancer, colorectal adenoma, cancer risk, colonoscopy, faecal immunochemical test (FIT)

## Abstract

**Aim:**

The COLO‐COHORT study aims to produce a multi‐factorial risk prediction model for colorectal neoplasia that can be used to target colonoscopy to those at greatest risk of colorectal neoplasia, ensuring that people are not investigated unnecessarily and maximizing the use of limited endoscopy resources. The study will also explore the link between neoplasia and the human gut microbiome. Additionally, the study aims to generate a cohort of colonoscopy patients who are ‘research ready’ through the development of a consent‐for‐contact (C4C) platform, to facilitate a range of colorectal cancer prevention studies to be conducted at scale and speed.

**Methods and analysis:**

This is a multi‐centre observational study involving sites across the UK. Recruitment is over a 6‐year period (2019–2025). Patients recruited to the study are those attending for colonoscopy. Patients are recruited into two groups, namely observational group A (10 000 patients) and C4C group B (10 000 patients), known as COLO‐SPEED (Colorectal Cancer Screening Prevention Endoscopy and Early Diagnosis; https://colospeed.uk). Patients complete a health questionnaire, provide anthropometric measurements and submit biosamples (blood and stool—depending on the part of the study they are recruited into). Patients' colonoscopy and histology findings are also recorded. Models of factors associated with the presence of neoplasia at colonoscopy will be developed using logistic or multinomial regression. For internal validation, model discrimination and calibration will be assessed and bootstrapping and cross‐validation approaches used. To enable long‐term follow‐up for outcomes related to colorectal cancer and polyps, patients are asked to consent to follow‐up through data linkage with national databases.

**Dissemination:**

In keeping with good research practice, following analysis by the study team the study investigators will make the anonymized dataset available to other researchers. The C4C platform will also be accessible to other researchers. The study findings will be submitted for publication in peer‐reviewed journals and lay summaries will be disseminated to participants and the wider public.

## INTRODUCTION

Colorectal cancer (CRC) is the second most common cause of cancer death worldwide accounting for 935 000 deaths per year [1]. In the UK around 42 000 people are diagnosed with CRC annually with 16 000 dying from it [2]. The majority of CRCs develop through well‐established pathways with pre‐cancerous colorectal lesions (colorectal adenomas and serrated polyps) progressing to CRC [3, 4]. The process can take 10–15 years and there is therefore a window of opportunity for these lesions to be detected and removed during colonoscopy [5].

Advances in prevention, diagnosis and management have resulted in an improvement in the mortality from CRC over the past few decades. In the UK, the introduction of the Bowel Cancer Screening Programme (BCSP) has led to a stage shift in CRC diagnosis; however, the majority of CRCs in the UK are still diagnosed through symptomatic services rather than screening [6].

In the UK more than 675 000 colonoscopies are performed annually and the demand is rising [7]. Endoscopy services were already struggling to provide capacity to meet this demand and the COVID‐19 pandemic has significantly reduced endoscopy capacity [8, 9, 10, 11]. For patients, colonoscopy may provoke anxiety, has some risk associated and requires pre‐procedural bowel preparation, which patients find unpleasant [12]. To best utilize this limited resource and ensure that people are not investigated unnecessarily, we need to better identify those at greatest risk of CRC and target colonoscopy at those individuals.

At present, the BCSP relies on one factor, age, as the only criterion for eligibility for screening. In England, Wales and Northern Ireland, individuals between 56 and 74 years are invited to participate in each nation's respective bowel cancer screening programme, with plans to reduce the age threshold to 50 years. In Scotland, 50 years of age is already used as the threshold for invitation to their programme. For symptomatic patients, referral for colonoscopy is largely guided by symptoms or clinical suspicion but symptoms do not correlate well with the presence of colorectal neoplasia [13]. A wide range of risk factors for CRC have been identified including increasing age, male sex, obesity, alcohol intake, smoking, ingestion of red meat, family history and reduced physical activity [14, 15, 16, 17]. Currently, these risk factors are not taken into account when assessing whether or not an individual may require a colonoscopy.

Biomarkers could also be of value for patient stratification for investigation. Non‐invasive tests such as the faecal immunochemical test (FIT) have been used successfully in the screening setting and have recently been introduced into the English BCSP, with use in the symptomatic setting evolving [18]. Two large, published UK cohorts report good diagnostic performance of the FIT in low‐risk patients in primary care [19, 20]. As a consequence of the reduction in availability of lower gastrointestinal services due to the COVID‐19 pandemic, in some areas the FIT has been used to prioritize patients for definitive lower gastrointestinal investigation; however, the use of the FIT varies hugely nationally.

Additionally, the relationship between gut microbiota and health and disease has been increasingly studied. There is evidence that certain micro‐organisms, in particular the *Fusobacterium* species, are associated with colorectal neoplasia; however, most existing studies are small and knowledge as to whether the gut microbiota could help identify or alter the natural history of patients who harbour the potential for colorectal neoplasia remains somewhat limited [21, 22, 23, 24].

Risk stratification is a technique for systematically categorizing patients based on their risk of a particular condition. Managing patients based on their risk level may make better use of limited health service resources whilst also benefitting patients by avoiding the need for unnecessary investigations in those at low risk [25]. This approach has been utilized in other areas of healthcare, for example in cardiovascular disease, using the QRISK score to guide the need for therapeutic prevention of vascular events (such as myocardial infarction and stroke) and the FIB‐4 score within hepatology to non‐invasively stage an individual's risk of fibrosis [26, 27]. The potential for using risk prediction models to identify patients with colorectal neoplasia has been increasingly studied. Various risk prediction models have been developed in both the screening and symptomatic settings but model performance varies [25, 28, 29]. Further work needs to be undertaken to achieve a risk model with sufficiently good performance for prediction of colorectal neoplasia to justify use in clinical practice. We hypothesize that it is possible to develop a risk prediction model using clinical factors and readily available laboratory biomarkers (including the FIT) that will enable us to predict patients at highest risk of colorectal neoplasia. We also hypothesize that the stool microbiome in patients may be helpful in identifying those at greatest neoplasia risk [30].

Most current research strategies are based on answering a single question with the study ending with the recruitment of the final patient; however, current good research practice supports making datasets discoverable [31]. Furthermore, most patients for CRC research are recruited on a study‐by‐study basis, despite it being advantageous to be able to deliver multiple studies simultaneously. An alternative approach is to develop a pool of research‐ready patients who can be contacted when studies relating to an aspect of screening, prevention and early diagnosis research relevant to them becomes available. This research‐ready consent‐for‐contact (C4C) population would enable a range of CRC‐related studies to be conducted at scale and speed and would facilitate rapid engagement with patients and the public in the development and design of research studies.

The objectives of the COLO‐COHORT study are as follows:

- to develop a multi‐factorial risk prediction model for prevalent colorectal neoplasia;
- to develop a cohort of patients who will be followed up long term through medical records and national databases for outcomes related to colorectal neoplasia, in order to test the long‐term value of the risk prediction model;
- to compare the structure and diversity of the faecal microbiome in patients with and without colorectal neoplasia;
- to develop a C4C platform of colonoscopy patients who have consented to be contacted for current and future research opportunities;
- to build a digital platform to support patient involvement, recruitment and data collection.

## METHODS AND ANALYSIS

### Study design

COLO‐COHORT is a multi‐centre observational study involving sites across the UK. Patients are recruited into two groups, namely group A and C4C group B, also called COLO‐SPEED (Colorectal Cancer Screening Prevention Endoscopy and Early Diagnosis; https://colospeed.uk). Recruitment will take place over a 6‐year period (2019–2025).

For group A, 10 000 patients will be recruited into the main observational element of the study. This group is subdivided into groups A1 and A2. Patients in group A1 submit blood and stool samples, whereas these samples are not required for patients in group A2; instead results from previous blood/stool tests are obtained from patient records.

For group B, 10 000 patients will be recruited into the C4C arm of the study (COLO‐SPEED). These patients may also be recruited into group A and/or into other endoscopy studies.

The study includes patients attending colonoscopy as part of the English BCSP and those referred through standard National Health Service (NHS) care for indications including, but not limited to, symptoms, family history or as part of surveillance programmes.

Groups A and B indicate what patients have consented to and are not defined to allow comparison between the groups.

### Study population: inclusion, exclusion and withdrawal criteria

Patients eligible for the study are those attending for a planned colonoscopy. Potential study participants are identified from outpatient clinics, endoscopy lists, pre‐assessment clinics or other clinical referral routes such as straight to test procedures depending upon how local services are configured. The approach to participant identification varies between sites due to variations in referral pathways to endoscopy and differing pre‐assessment approaches; therefore, where local pathways differ, these are incorporated.

#### Group A

##### Inclusion criteria

- Aged ≥30 years and able to give informed consent (colorectal neoplasia is very rare in people below 30 years and this is why this age threshold was chosen);
- Patients attending colonoscopy:
  - through the BCSP (FIT positive, Bowelscope conversion, surveillance);
  - through standard NHS care (most commonly referred due to iron deficiency anaemia, altered bowel habit, weight loss, rectal bleeding, planned polypectomy, on the basis of family history, abnormal cross‐sectional imaging, polyp surveillance or post CRC surveillance).

##### Exclusion criteria

- Unable to give informed consent;
- Known polyposis syndrome;
- Previous total colectomy;
- Known colonic stricture which would limit complete colonoscopy;
- Attending for planned therapeutic procedure other than polypectomy, such as insertion of colonic stent;
- Attending for assessment of known inflammatory bowel disease activity or for inflammatory bowel disease surveillance;
- Patients currently recruited into an interventional clinical trial of a medicinal product for CRC prevention.

#### Group B

The COLO‐SPEED funding infrastructure is currently only available to sites that are part of the Northern Region Endoscopy Group (NREG.org.uk) and thus only patients from the northeast of England can be recruited into group B.

##### Inclusion criteria

- Any patient attending for colonoscopy and able to give informed consent;
- ≥18 years old;
- Patient attending for colonoscopy in a site supported by COLO‐SPEED infrastructure.

##### Exclusion criteria

- Unable to give informed consent.

COLO‐SPEED (group B) aims to establish a C4C database; therefore participation in parallel research studies is encouraged and not an exclusion criterion.

##### Withdrawal criteria

Patients from either group A or group B are withdrawn from the study if they withdraw consent for study participation or withdraw consent to undergo colonoscopy.

### Recruitment process

All eligible and consenting patients are recruited following referral for colonoscopy. COLO‐COHORT commenced just before the COVID‐19 pandemic and, in response to the pandemic, the recruitment process was adapted to minimize the time patients spend in the hospital for research purposes. Patients are contacted via telephone prior to their colonoscopy appointment to assess interest in study participation. If patients are interested in the study, the research team send out a patient information sheet and a FIT (as applicable) and arrange to contact the patient via telephone again at a later date. A pre‐bowel preparation FIT sample is required for the study and therefore those patients who are required to submit a new FIT sample need to be contacted in a timeframe that allows for this. To facilitate the return of FIT samples, patients are provided with a labelled postage paid return envelope. Alternatively, patients can return their FIT to the research team on the day of their colonoscopy.

Patients undergoing a colonoscopy as part of the BCSP are not sent a FIT as this has already been undertaken as part of the screening programme. The same FIT used within the English BCSP (OC Sensor, Mast Diagnostics) is provided to symptomatic patients for uniformity of quantitative FIT results as FIT varies between different manufacturers [32]. Where patients have already undertaken a FIT as part of a symptomatic pathway, the FIT is repeated in this study. The FIT results generated within the study are not directly used to inform patient management through the study; however, local principal investigators are informed of abnormal results and have discretion to act upon these where there is clinical concern.

On the second contact, a member of the research team discusses the study with the patient, answers any questions, assesses eligibility, obtains verbal consent (if the patient is eligible and willing to take part) and gathers other information required for the study.

On the day of the colonoscopy appointment, written informed consent is obtained, anthropometric measurements and blood tests (as applicable per study group) are taken, and patients are recruited into the study.

The adaptations to minimize face‐to‐face contact in response to the COVID‐19 pandemic will be reviewed on an ongoing basis as the pandemic changes and face‐to‐face contact will be reinstated if and where it is considered appropriate.

### Data collection

#### Group A

**FIGURE 1:**
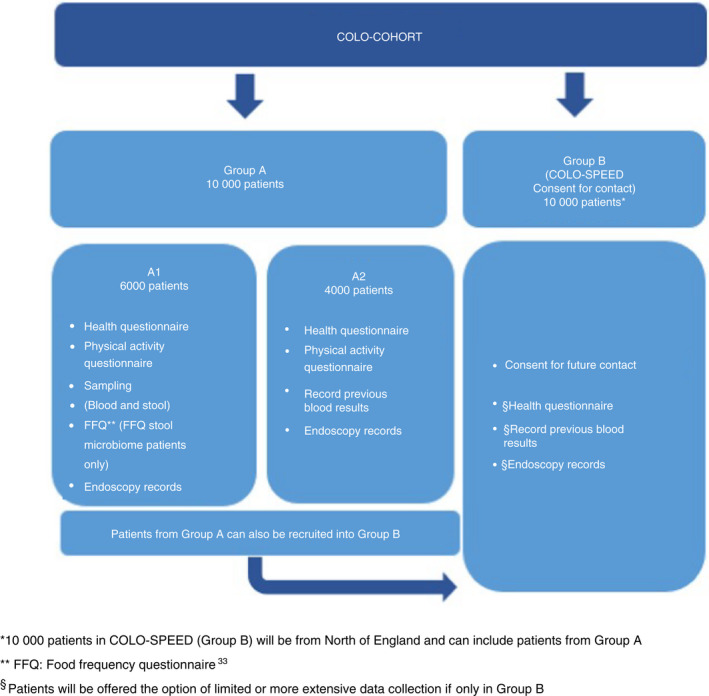
Overview of COLO‐COHORT. *10 000 patients in COLO‐SPEED (group B) will be from the north of England and can include patients from group A. **FFQ, food frequency questionnaire [33]. ^§^Patients will be offered the option of limited or more extensive data collection if only in group B.

Six thousand participants in group A (group A1) will submit a pre‐bowel preparation FIT sample. This will be a combination of new FIT collections and samples from patients who have already submitted a FIT sample as part of the BCSP (Figure 1).

As approved by the ethics committee, submission of the FIT sample represents initial consent for the study with further information provided in the patient information sheet and full written consent given on the day of colonoscopy. All FIT samples are returned to the northeast BCSP hub at Queen Elizabeth Hospital, Gateshead, for analysis. For patients recruited who are attending colonoscopy because of a positive FIT taken in the BCSP, the quantitative result of that FIT is transferred to the study database.

In addition, a health questionnaire detailing personal characteristics (level of education, employment status), lifestyle behaviour (smoking status and alcohol intake history), medical and medication history, and family history of CRC is completed, as well as a validated physical activity questionnaire and validated food frequency questionnaire for those undergoing microbiome analysis [33, 34]. In the light of the COVID‐19 pandemic, questionnaires may be completed remotely from the endoscopy unit. Postage paid envelopes are provided by the research team to facilitate the return of completed questionnaires.

At the colonoscopy appointment, height, weight and waist circumference are measured. In addition, blood samples for full blood count, liver function tests, aspartate aminotransferase, C‐reactive protein, lipid profile, fasting glucose, HbA1c, and whole blood for DNA extraction are taken, via a cannula inserted as part of standard care for colonoscopy. Where sampling is not possible from a cannula, a separate venepuncture is undertaken. These tests are not repeated if results from within the last 8 weeks are available. All blood tests other than the blood sample for DNA extraction are labelled and processed in line with local Trust policy in local laboratories. The sample for DNA is pseudonymized using a unique study ID and transferred to the Central Biobank Facility at Newcastle University. Samples are stored at −80°C until thawed in preparation for DNA extraction; following extraction genomic DNA is stored at −80°C.

Patients' colonoscopy findings are recorded along with histopathology results from any lesion removed or sampled. Diagnosis of neoplasia is based upon histological findings.

A further 4000 patients will be recruited into group A (group A2). Although the dataset recorded is similar to group A1, patients are not required to collect stool for the FIT nor have blood samples taken. This allows patients who are unable or unwilling to provide biosamples to participate in this part of the study. These patients will provide the research team with a significant amount of useful data to enhance or potentially validate data from the A1 group. Any recent blood tests of interest in the past 8 weeks prior to their colonoscopy (as a minimum, full blood count and liver function tests) as well as FIT results if done as part of routine care are recorded.

All patients from group A are asked if they also consent to the following:

- long‐term follow‐up for future outcome related to colorectal polyps or CRC through linkage to routine national databases such as National Cancer Registration Database, Hospital Episode Statistics data, Office for National Statistics mortality data, National Endoscopy Database and the COloRECTal cancer data Repository (CORECT‐R) [35];
- access to previous endoscopy results, histological samples from colonoscopy or other relevant laboratory results;
- use of anonymized information or samples collected from this study in future studies;
- consent for future contact for collection of additional information related to this study.

Patients may opt into or out of each of these.

### Microbiome

Stool microbiome analysis is performed using the stool sample submitted for the FIT. The rationale for this is based upon our pilot work that demonstrated excellent agreement with fresh stool analysis and the stability of microbiome diversity and taxonomic profiles when using the FIT test kit (OC Sensor, Mast Diagnostics) for microbiome analysis [36].

Microbiome analysis is performed on all new FIT collections and (subject to patient consent) on positive BCSP FIT samples that were analysed by the northeast BCSP hub. Patients undergoing microbiome testing are asked to complete the EPIC food frequency questionnaire for dietary information [33].

Once the FIT is performed, the pseudonymized samples (labelled with unique study ID) are transferred to the Central Biobank Facility at Newcastle University. Samples are frozen (at −80°C) on arrival at the Biobank Facility; batched samples are then thawed and isolation of faecal DNA is performed using the DNeasy PowerLyzer PowerSoil Kit (QIAGEN). The QIAcube HT provides automation for this process and facilitates the extraction of faecal DNA in a timely manner. Microbiome analysis will then be performed at the University of Leeds and Newcastle University.

Stool microbiome analysis is a rapidly evolving field [21, 22]. In the first instance, we propose to measure microbiome diversity and individual pathobionts by 16S rRNA sequencing but will remain flexible with our approach to, for example, shotgun metagenomic sequencing based on subsequent developments in the field, DNA yields from FIT stool extractions and our funding envelope [36, 37] (Table 1).

**TABLE 1:** Summary of data collection for group A

Questionnaire	Measurement	Sampling	Colonoscopy
Health questionnaire Physical activity questionnaire FFQ^a^	Height Weight Waist circumference	Stool for FIT^b^ Blood tests	Colonoscopy report Histology report

Abbreviations: FFQ, food frequency questionnaire; FIT, faecal immunochemical test.

^a^ Patients undergoing microbiome analysis only.

^b^ Non Bowel Cancer Screening Programme patients.

#### Group B (C4C; COLO‐SPEED)

Patients from group A can also be recruited into group B.

Patients are recruited to COLO‐SPEED from northeastern recruitment sites, with the longer‐term vision of expanding to other sites nationally, subject to securing funding.

All patients consenting to group B consent to being contacted about future research involvement opportunities, including relevant research studies, patient and public involvement (PPI) opportunities and public engagement activities. They will be sent a link to register online for the COLO‐SPEED Research Network (CSRN).

In addition to consent for future contact, patients are asked if they wish to consent to the following:

- long‐term follow‐up for future outcome related to colorectal polyps or CRC through linkage to routine national databases such as the National Cancer Registration Database, Hospital Episode Statistics data, Office for National Statistics mortality data, National Endoscopy Database and the CORECT‐R [35];
- access to previously collected endoscopy results, histological samples from colonoscopy or other relevant laboratory results;
- use of anonymized information or samples collected from this study in future studies;
- consent for future contact for collection of additional information related to this study.

Group B patients are offered the choice of limited data collection or more extensive data collection. Limited data collection includes their age, sex, ethnicity, family history, height and weight measurements, indication for colonoscopy, and colonoscopy and histology results (where applicable). More extensive collection comprises, in addition to the elements of the limited collection, the health questionnaire and the recording of recent blood results (within the past 8 weeks) and/or FIT results if available.

No new samples (blood or stool) are taken from COLO‐SPEED (group B) patients unless they are also part of group A.

### Nested studies

COLO‐COHORT will recruit a large population, and appropriate nested studies, for example collecting data on patient experience of colonoscopy or impact of COVID‐19 on colonoscopy uptake and experience, may be incorporated into the study (subject to additional ethical approval where required).

### Adverse events

This is an observational study and therefore no adverse events resulting from participation are anticipated. Any adverse events related to colonoscopy will be managed and recorded in line with standard care.

### Assessment and follow‐up

No additional study visits are required as part of the study. However, patients may receive a maximum of two reminders at monthly intervals to complete the patient questionnaires (outlined above), if required.

Patients who consent to long‐term follow‐up may have their endoscopy records and medical records interrogated for outcomes related to colorectal neoplasia as described above.

### Statistical analysis and sample size

Data from patients recruited to group A will be used to develop the risk prediction model. Primary analysis will be based on the outcome of the presence of colorectal neoplasia at the recruitment colonoscopy. This will be defined as the presence of advanced adenoma (AA)/CRC. AA will be defined as an adenoma of at least 10 mm in size or containing high‐grade dysplasia [38]. Secondary analyses will focus on other relevant outcomes, including CRC (only), AA (only), any adenoma, any polyp, serrated polyps, numbers of adenomas and number of polyps. The TRIPOD statement will be followed for reporting of the risk prediction model [39].

Initially we will develop models separately for BCSP and other (mainly symptomatic) subjects; further analysis will explore whether an overall model can be created. Firstly (in the non‐BCSP subjects), sensitivity, specificity, positive predictive value and negative predictive value of FIT alone for the detection of the presence of AA/CRC will be assessed. Different FIT cut‐offs will be explored. Subsequently, the relationship between patient characteristics and lifestyle, phenotypic information, FIT results, blood markers and presence of AA/CRC will be investigated using logistic regression. Backwards elimination of candidate predictors will be used to identify variables which best predict neoplasia [40]. Receiver operating curve plots, area under the curve analyses of sensitivity and specificity, and Harrell's C‐index will be used to characterize the discriminative ability of the models [41, 42]. Calibration will be assessed using Hosmer and Lemeshow tests, the percentage of people reclassified by different models and the net reclassification index [43]. Internal validation will be undertaken using bootstrapping to quantify the model's potential for overfitting and optimism in estimated model performance [40]. Cross‐validation approaches will also be applied, systematically excluding groups of subjects (e.g., by site) in turn [44]. In addition, multinomial, or ordinal, logistic regression will be used to investigate predictors of the secondary outcomes. Multiple adenomas may occur in an individual. It is anticipated that the presence of multiple adenomas will be zero‐inflated, with some patients having zero and others many. The extent to which zero‐inflated models (e.g., allowing for aggregation) improve the prediction of the presence of adenomas will be assessed.

The microbiome data will be subjected to canonical correspondence analysis to identify (i) major trends in variation across the patient cohort and (ii) putative drivers of the variation in each patient subgroup (defined on the basis of colonoscopy result: CRC, advanced adenomas, non‐advanced adenomas, sessile serrated polyps and clear colon). Canonical correspondence analysis seeks to explain the pattern of variation in complex (multivariate) datasets using covariates hypothesized to be of significance in causing variation. We hypothesize that there will be differences in microbiome composition that will be dependent on the adenoma/disease status of patients and that these may act as markers for disease. We will use similar approaches to determine whether dietary data, when combined with the microbiome, reveal further differences between groups. Correspondence analysis will be used to provide summaries of the variation microbiome to allow for investigation of the contribution of microbiota profile to the risk prediction models.

The study is exploratory in nature—particularly in relation to whether microbiome data make an important contribution to risk prediction models. Therefore the sample size calculation must be governed by principles of adequate population size.

The sample size of patients providing biosamples (6000) is based on the power calculation by Peduzzi et al. [45]: sample size *N* = 10(*k*/*p*), where *k* is the number of covariates to be investigated in the model and *p* is the smallest of the proportions of positive or negative cases.

Using Demidenko's method, the sample size needed for logistic regression with 80% power and 5% level of significance is *N* = 8*V*/*β*2, where *β* is the natural log of the odds ratio and *V* is logistic model variance due to covariates [46]. This formula can be rearranged to determine the minimum odds ratio that can be detected at a sample size of 6000 with sufficient power. Assuming a moderate standard error in the regression (SE = 0.125) and calculating *V* from the standard error (*V* = (SE√*N*)2) the study will have sufficient statistical power to detect an effect with a small effect size with an odds ratio of 1.13.

Group B sample size is based upon generating a significant but manageable C4C population.

A full statistical analysis plan will be developed prior to data analysis.

### Study governance and dissemination

#### Study oversight

To provide robust governance and monitoring, a three‐tiered governance structure has been devised. The COLO‐COHORT Central Study Delivery Team meet weekly and deliver the study and undertake regular study monitoring for sites. The Central Study Delivery Team is supervised by the study Oversight Group, composed of the study co‐investigators who have monthly contact to monitor study progress. Lastly, the study Advisory Group, made up of experts in stool microbiome and in management of large study databases, provide scientific, clinical and research advice to inform direction of the study via annual meetings (Table 2).

**TABLE 2:** COLO‐COHORT governance structure and frequency of meetings

Central Study Delivery Team	Oversight Group	Advisory Group
Weekly meetings	Monthly teleconference meetings Two face‐to‐face meetings per year	Annual meetings (teleconference or face‐to‐face)

### Patient and public involvement (PPI)

Patients and the public are extensively involved in this study, including in the development of procedures and review of patient facing materials. A patient representative attends regular COLO‐COHORT research group meetings and is a co‐author of this paper. Several workshops and PPI days have been organized to discuss and improve the study. Sessions will be organized to feed back and discuss study results.

### Data storage and management

Data collected are recorded onto REDCap, a secure online database [47, 48]. Data are pseudonymized with each patient's unique study ID.

The personal identifiable data required for the C4C patient group (COLO‐SPEED, group B) is held in each local recruiting site and subsequently will be sent to the Newcastle University research team, via secure encrypted email, who will send participants the link for registration and profile creation to the CSRN, after which all personal identifiable data will be securely destroyed.

In keeping with good research practice, the study co‐investigators will make the anonymized dataset available to other researchers. How to apply for this will be made available on the study website in due course (https://colospeed.uk). The platform is also available to researchers wishing to utilize this research‐ready population to increase recruitment to and engagement with new studies. Access to this will be granted following review by the study co‐investigators and study management group.

### Dissemination

For academic and clinical dissemination, the results will be submitted for publication in high‐impact international peer‐reviewed journals and presented to scientific meetings. Additionally, to support and promote PPI, lay summaries will be prepared and posted on the study website. The study team will also work with the PPI representative to identify other routes for lay dissemination.

To maximize clinical impact, the research team will actively seek to work with other datasets/researchers to undertake external validation of the risk prediction model.

## DISCUSSION

Development of risk adapted triage for colonoscopy has been identified as a research priority [49]. COLO‐COHORT will produce a multi‐factorial risk prediction model for colorectal neoplasia based upon a large cohort of patients attending for colonoscopy at sites with diverse catchment populations. Following external validation, this model can then be used to inform more intelligent colonoscopy, directing limited endoscopy resources to those individuals at highest risk and additionally avoiding unnecessary investigations for those at low risk. It is acknowledged that several recent studies have demonstrated the value of the FIT in CRC risk prediction, but this study goes significantly further by looking more widely at neoplasia and considering factors not included in many of the previous FIT studies, notably the microbiome. As risk models are developed, they have potential to be applied to other datasets for validation.

The study will provide large‐scale gut microbiota analysis in both symptomatic and screening patients aiding the understanding of the role of the gut microbiome and its association with colorectal neoplasia. The study will adopt the large‐scale use of FIT samples for microbiome analysis. The FIT is used widely, within the national BCSP and with increasing use in the symptomatic setting. The wide availability of these samples has cost saving implications for the study (and for future application of the approach) and increased convenience for patients, with only one sample required [36].

COLO‐COHORT will also provide a large C4C platform which will allow current concurrent research to be undertaken, for example testing of patient experience of colonoscopy, provide large datasets for study and provide a population of research‐ready patients who can be contacted for future research. Upon registration for the CSRN, participants will be able to indicate the types of research areas and opportunities they would be happy to receive information about in the future. Research opportunities include invitations to public engagement events, research news, PPI activities and relevant research studies for which they may be eligible. The C4C approach has been used by the Join Dementia Research initiative where individuals are encouraged to sign up for potential dementia‐based studies (www.joindementiaresearch.nihr.ac.uk) [50]. COLO‐COHORT provides the first colorectal C4C initiative and has the advantage that the neoplasia status of all individuals is already known when they enter the C4C platform (as they are all attending for colonoscopy). We believe that this will provide an innovative, unique and invaluable resource for CRC researchers, right across the disease pathway from prevention to survival and survivorship.

The COLO‐COHORT study has adopted a recruitment approach that allows patients to choose the extent of their involvement whilst maximizing recruitment numbers through offering different opportunities. The study will be adapted where necessary to maximize recruitment and use of resources. For example, the approach to recruitment within the COLO‐COHORT was adapted in view of the COVID‐19 pandemic with ‘remote’ contact maximized to avoid additional time in hospital for research purposes. This approach was favoured by patients and allowed ongoing recruitment during the pandemic.

As far as we are aware, COLO‐SPEED will be the first ever C4C research platform for patients being investigated for potential CRC. This platform will facilitate future research to be conducted at speed and scale. This will allow more rapid delivery of research and thus more rapid translation of results into patient benefit.

## ETHICS STATEMENT

The study was reviewed and approved by the West Midlands Edgbaston Research Ethics Committee (June 2019) and received favourable Bowel Cancer Screening Programme Research Advisory Committee approval (October 2019).

## CONFLICT OF INTEREST

CJR has received grant funding from ARC Medical, Norgine, Medtronic, 3D Matrix Solutions and Olympus Medical. He was an expert witness for ARC Medical. LS is in receipt of project grants from 3D Matrix and Medtronic. The remaining authors declare no competing interests.

## AUTHOR CONTRIBUTIONS

JSH: contributed to design and delivery of the study; authored the first draft of the manuscript and approved submitted version. SK: contributed to design and delivery of the study; contributed to the manuscript and approved submitted version. CD: contributed to the design and delivery of the study, revised draft manuscript, approved submitted version. CS: contributed to design for FIT sampling, storage and DNA extractions, revised draft manuscript, approved submitted version. LJN: contributed to design and delivery of the study, revised draft manuscript and approved submitted version. KM: contributed to design of the digital work, revised draft manuscript and approved submitted version. SM: provided expertise in the design and conduct of microbiome analyses, revised draft manuscript, approved submitted version. JW: contributed to the development and reviewed all patient facing documents. CA: contributed to study design and delivery, revised draft manuscript and approved submitted version. PK: contributed to study design and delivery, revised draft manuscript and approved submitted version. SR: contributed to study design, revised draft manuscript and approved submitted version. MAH: co‐developed the idea and plans for the study; secured funding; provided expertise in design and conduct of the sample biobank and laboratory analyses; revised draft manuscript; approved submitted version. LS: co‐developed the idea and plans for the study; secured funding; provided epidemiological expertise in design and conduct; revised draft manuscript; approved submitted version. CJR: co‐developed the idea and plans for the study; secured funding; provided clinical expertise in design and conduct; revised draft manuscript; approved submitted version and oversaw authorship of manuscript.

## Data Availability

In keeping with good research practice, following analysis by the study team the study investigators will make the anonymised dataset available to other researchers.

## References

[1] Sung H , Ferlay J , Siegel RL , Laversanne M , Soerjomataram I , Jemal A , et al. Global cancer statistics 2020: GLOBOCAN estimates of incidence and mortality worldwide for 36 cancers in 185 countries. CA Cancer J Clin. 2021;71(3):209–49. 10.3322/caac.21660 33538338

[2] Cancer Research UK . Bowel cancer statistics. [Cited 2019 Dec 8]. Available from: https://www.cancerresearchuk.org/health‐professional/cancer‐statistics/statistics‐by‐cancer‐type/bowel‐cancer#heading‐Zero

[3] Leslie A , Carey FA , Pratt NR , Steele RJC . The colorectal adenoma–carcinoma sequence. Br J Surg. 2002;89(7):845–60. 10.1046/j.1365-2168.2002.02120.x 12081733

[4] East JE , Atkin WS , Bateman AC , Clark SK , Dolwani S , Ket SN , et al. British Society of Gastroenterology position statement on serrated polyps in the colon and rectum. Gut. 2017;66(7):1181–96. 10.1136/gutjnl-2017-314005 28450390 PMC5530473

[5] Day DW . The adenoma–carcinoma sequence. Scand J Gastroenterol Suppl. 1984;104:99–107. 6597553

[6] Gill MD , Bramble MG , Rees CJ , Lee TJW , Bradburn DM , Mills SJ . Comparison of screen‐detected and interval colorectal cancers in the Bowel Cancer Screening Programme. Br J Cancer. 2012;107(3):417–21. 10.1038/bjc.2012.305 22782347 PMC3405230

[7] Centre for Workforce Intelligence. Securing the future workforce supply: gastrointestinal endoscopy workforce Review; 2017.

[8] Ravindran S , Bassett P , Shaw T , Dron M , Broughton R , Johnston D , et al. National census of UK endoscopy services in 2019. Frontline Gastroenterol. 2020;12(6):flgastro‐2020‐101538. 10.1136/flgastro-2020-101538 PMC8515281 34712462

[9] Rees CJ , East JE , Oppong K , Veitch A , McAlindon M , Anderson J , et al. Restarting gastrointestinal endoscopy in the deceleration and early recovery phases of COVID‐19 pandemic: guidance from the British Society of Gastroenterology. Clin Med (Northfield Il). 2020;20(4):352–8. 10.7861/clinmed.2020-0296 PMC7385767 32518104

[10] Maringe C , Spicer J , Morris M , Purushotham A , Nolte E , Sullivan R , et al. The impact of the COVID‐19 pandemic on cancer deaths due to delays in diagnosis in England, UK: a national, population‐based, modelling study. Lancet Oncol. 2020;21:1023–34. 10.1016/S1470-2045(20)30388-0 32702310 PMC7417808

[11] Rutter MD , Brookes M , Lee TJ , Rogers P , Sharp L . Impact of the COVID‐19 pandemic on UK endoscopic activity and cancer detection: a National Endoscopy Database Analysis. Gut. 2021;70(3):537–43. 10.1136/gutjnl-2020-322179 32690602

[12] Neilson LJ , Patterson J , von Wagner C , Hewitson P , McGregor LM , Sharp L , Rees CJ . Patient experience of gastrointestinal endoscopy: informing the development of the Newcastle ENDOPREM™. Frontline Gastroenterol. 2020;11(3):209–17. flgastro‐2019‐101321. 10.1136/flgastro-2019-101321 32419912 PMC7223270

[13] Adelstein B‐A , Macaskill P , Chan SF , Katelaris PH , Irwig L . Most bowel cancer symptoms do not indicate colorectal cancer and polyps: a systematic review. BMC Gastroenterol. 2011;11:65. 10.1186/1471-230X-11-65 21624112 PMC3120795

[14] Harriss DJ , Atkinson G , George K , Tim Cable N , Reilly T , Haboubi N , et al. Lifestyle factors and colorectal cancer risk (1): systematic review and meta‐analysis of associations with body mass index. Colorectal Dis. 2009;11(6):547–63. 10.1111/j.1463-1318.2009.01766.x 19207714

[15] Okabayashi K , Ashrafian H , Hasegawa H , Yoo JH , Patel VM , Harling L , et al. Body mass index category as a risk factor for colorectal adenomas: a systematic review and meta‐analysis. Am J Gastroenterol. 2012;107:1175–85. 10.1038/ajg.2012.180 22733302

[16] Johnson CM , Wei C , Ensor JE , Smolenski DJ , Amos CI , Levin B , et al. Meta‐analyses of colorectal cancer risk factors. Cancer Causes Control. 2013;24(6):1207–22. 10.1007/s10552-013-0201-5 23563998 PMC4161278

[17] World Cancer Research Fund International/American Institute for Cancer Research . Continous Update Project Expert Report 2018. Diet, Nutrition, Physical Activity and Colorectal Cancer. [Cited 2021 Jun 20]. Available from: https://www.wcrf.org/wp‐content/uploads/2021/02/Colorectal‐cancer‐report.pdf

[18] Westwood M , Lang S , Armstrong N , Van Turenhout S , Cubiella J , Stirk L , et al. Faecal immunochemical tests (FIT) can help to rule out colorectal cancer in patients presenting in primary care with lower abdominal symptoms: a systematic review conducted to inform new NICE DG30 diagnostic guidance. BMC Med. 2017;15(1):189. 10.1186/s12916-017-0944-z 29061126 PMC5654140

[19] Bailey SER , Abel GA , Atkins A , Byford R , Davies SJ , Mays J , et al. Diagnostic performance of a faecal immunochemical test for patients with low‐risk symptoms of colorectal cancer in primary care: an evaluation in the south west of England. Br J Cancer. 2021;124:1231–6. 10.1038/s41416-020-01221-9 33462361 PMC8007716

[20] Nicholson BD , James T , Paddon M , Justice S , Oke JL , East JE , et al. Faecal immunochemical testing for adults with symptoms of colorectal cancer attending English primary care: a retrospective cohort study of 14 487 consecutive test requests. Aliment Pharmacol Ther. 2020;52:1031–41. 10.1111/apt.15969 32677733

[21] Cheng Y , Ling Z , Li L . The intestinal microbiota and colorectal cancer. Front Immunol. 2020;11:3100. 10.3389/fimmu.2020.615056 PMC7734048 33329610

[22] Mima K , Kosumi K , Baba Y , Hamada T , Baba H , Ogino S . The microbiome, genetics, and gastrointestinal neoplasms: the evolving field of molecular pathological epidemiology to analyze the tumor–immune–microbiome interaction. Hum Genet. 2021;140(5):725–46. 10.1007/s00439-020-02235-2 33180176 PMC8052267

[23] Flemer B , Lynch DB , Brown JMR , Jeffery IB , Ryan FJ , Claesson MJ , et al. Tumour‐associated and non‐tumour‐associated microbiota in colorectal cancer. Gut. 2017;66(4):633–43. 10.1136/gutjnl-2015-309595 26992426 PMC5529966

[24] Thomas AM , Manghi P , Asnicar F , Pasolli E , Armanini F , Zolfo M , et al. Metagenomic analysis of colorectal cancer datasets identifies cross‐cohort microbial diagnostic signatures and a link with choline degradation. Nat Med. 2019;25(4):667–78. 10.1038/s41591-019-0405-7 30936548 PMC9533319

[25] Hull MA , Rees CJ , Sharp L , Koo S . A risk‐stratified approach to colorectal cancer prevention and diagnosis. Nat Rev Gastroenterol & Hepatol. 2020;17(12):773–80. 10.1038/s41575-020-00368-3 33067592 PMC7562765

[26] Hippisley‐Cox J , Coupland C , Brindle P . Development and validation of QRISK3 risk prediction algorithms to estimate future risk of cardiovascular disease: prospective cohort study. BMJ. 2017;357:j2099. 10.1136/bmj.j2099 28536104 PMC5441081

[27] Vallet‐Pichard A , Mallet V , Nalpas B , Verkarre V , Nalpas A , Dhalluin‐Venier V , et al. FIB‐4: an inexpensive and accurate marker of fibrosis in HCV infection. Comparison with liver biopsy and fibrotest. Hepatology. 2007;46(1):32–6. 10.1002/hep.21669 17567829

[28] Usher‐Smith JA , Walter FM , Emery JD , Win AK , Griffin SJ . Risk prediction models for colorectal cancer: a systematic review. Cancer Prev Res (Phila). 2016;9(1):13–26. 10.1158/1940-6207.CAPR-15-0274 26464100 PMC7610622

[29] Williams TGS , Cubiella J , Griffin SJ , Walter FM , Usher‐Smith JA . Risk prediction models for colorectal cancer in people with symptoms: a systematic review. BMC Gastroenterol. 2016;16(1):63. 10.1186/s12876-016-0475-7 27296358 PMC4907012

[30] Loke YL , Chew MT , Ngeow YF , Lim WWD , Peh SC . Colon carcinogenesis: the interplay between diet and gut microbiota. Front Cell Infect Microbiol. 2020;10:603086. 10.3389/fcimb.2020.603086 33364203 PMC7753026

[31] Centre for Open Science . Open Science Framework. [Cited 2021 Jun 20]. Available from: https://www.cos.io/about/mission

[32] Chapman CJ , Banerjea A , Humes DJ , Allen J , Oliver S , Ford A , et al. Choice of faecal immunochemical test matters: comparison of OC‐sensor and HM‐JACKarc, in the assessment of patients at high risk of colorectal cancer. Clin Chem Lab Med. 2020;59(4):721–8. 10.1515/cclm-2020-1170 33112776

[33] EPIC Norfolk . The EPIC‐Norfolk Food Frequency Questionnaire and FETA Software

[34] MRC Epidemiology Unit . EPIC Physical Activity Questionnaire.

[35] UK Colorectal Cancer Intelligence Hub . COloRECTal cancer data Repository (CORECT‐R). https://www.ndph.ox.ac.uk/corectr

[36] Masi AC , Koo S , Lamb CA , Hull MA , Sharp L , Nelson A , et al. Using faecal immunochemical test (FIT) undertaken in a national screening programme for large‐scale gut microbiota analysis. Gut. 2021;70:429–431. 10.1136/gutjnl-2020-321594 32430347

[37] Watson H , Mitra S , Croden FC Croden FC , Taylor M , Wood HM , Perry SL , Spencer JA , Quirke P , Toogood GJ , Lawton CL , Dye L , Loadman PM , Hull MA A randomised trial of the effect of omega‐3 polyunsaturated fatty acid supplements on the human intestinal microbiota. Gut. 2018;67(11):1974–83. 10.1136/gutjnl-2017-314968 28951525

[38] Rutter MD , East J , Rees CJ , Cripps N , Docherty J , Dolwani S , et al. British Society of Gastroenterology/Association of Coloproctology of Great Britain and Ireland/Public Health England post‐polypectomy and post‐colorectal cancer resection surveillance guidelines. Gut. 2020;69(2):201–23. 10.1136/gutjnl-2019-319858 31776230 PMC6984062

[39] Collins GS , Reitsma JB , Altman DG , Moons KGM . Transparent reporting of a multivariable prediction model for individual prognosis or diagnosis (TRIPOD): the TRIPOD statement. Br Med J. 2015;350:g7594. 10.1136/bmj.g7594 25569120

[40] Moons KGM , Kengne AP , Woodward M , Royston P , Vergouwe Y , Altman DG , et al. Risk prediction models: I. Development, internal validation, and assessing the incremental value of a new (bio)marker. Heart. 2012;98(9):683–90. 10.1136/heartjnl-2011-301246 22397945

[41] Cook NR . Statistical evaluation of prognostic versus diagnostic models: beyond the ROC curve. Clin Chem. 2008;54(1):17–23. 10.1373/clinchem.2007.096529 18024533

[42] Harrell FE , Lee KL , Mark DB . Multivariable prognostic models: issues in developing models, evaluating assumptions and adequacy, and measuring and reducing errors. Stat Med. 1996;15(4):361–87. 10.1002/(sici)1097-0258(19960229)15:4 8668867

[43] Pencina MJ , D'Agostino RB , Steyerberg EW . Extensions of net reclassification improvement calculations to measure usefulness of new biomarkers. Stat Med. 2011;30(1):11–21. 10.1002/sim.4085 21204120 PMC3341973

[44] Van Heijningen EMB , Lansdorp‐Vogelaar I , Van Hees F , Kuipers EJ , Biermann K , de Koning HJ , et al. Developing a score chart to improve risk stratification of patients with colorectal adenoma. Endoscopy. 2016;48:563–70. 10.1055/s-0042-104275 27167762

[45] Peduzzi P , Concato J , Kemper E , Holford TR , Feinstein AR . A simulation study of the number of events per variable in logistic regression analysis. J Clin Epidemiol. 1996;49(12):1373–9. 10.1016/S0895-4356(96)00236-3 8970487

[46] Demidenko E . Sample size determination for logistic regression revisited. Stat Med. 2007;26:3385–97. 10.1002/sim.2771 17149799

[47] Harris PA , Taylor R , Thielke R , Payne J , Gonzalez N , Conde JG . Research electronic data capture (REDCap)—a metadata‐driven methodology and workflow process for providing translational research informatics support. J Biomed Inform. 2009;42(2):377–81. 10.1016/j.jbi.2008.08.010 18929686 PMC2700030

[48] Harris PA , Taylor R , Minor BL , Elliott V , Fernandez M , O'Neal L , et al. The REDCap consortium: building an international community of software platform partners. J Biomed Inform. 2019;95:103208. 10.1016/j.jbi.2019.103208 31078660 PMC7254481

[49] Lawler M , Alsina D , Adams RA , Anderson AS , Brown G , Fearnhead NS , et al. Critical research gaps and recommendations to inform research prioritisation for more effective prevention and improved outcomes in colorectal cancer. Gut. 2018;67(1):179–93. 10.1136/gutjnl-2017-315333 29233930 PMC5754857

[50] Join Dementia Research . Welcome to Join Dementia Research. [Cited 2022 Feb 28]. Available from: https://www.joindementiaresearch.nihr.ac.uk/

